# Current understanding of IgA antibodies in the pathogenesis of IgA nephropathy

**DOI:** 10.3389/fimmu.2023.1165394

**Published:** 2023-04-11

**Authors:** Yoshihito Nihei, Hitoshi Suzuki, Yusuke Suzuki

**Affiliations:** ^1^ Department of Nephrology, Juntendo University Faculty of Medicine, Tokyo, Japan; ^2^ Department of Nephrology, Juntendo University Urayasu Hospital, Chiba, Japan

**Keywords:** IgA, IgA nephropathy, galactose deficient IgA1, immune complex, pathogenesis

## Abstract

Immunoglobulin A (IgA) is the most abundant isotype of antibodies, provides a first line of defense at mucosal surfaces against pathogens, and thereby contributes to mucosal homeostasis. IgA is generally considered as a non-inflammatory antibody because of its main function, neutralizing pathogenic virus or bacteria. Meanwhile, IgA can induce IgA-mediated diseases, such as IgA nephropathy (IgAN) and IgA vasculitis. IgAN is characterized by the deposition of IgA and complement C3, often with IgG and/or IgM, in the glomerular mesangial region, followed by mesangial cell proliferation and excessive synthesis of extracellular matrix in glomeruli. Almost half a century has passed since the first report of patients with IgAN; it remains debatable about the mechanism how IgA antibodies selectively bind to mesangial region—a hallmark of IgAN—and cause glomerular injuries in IgAN. Previous lectin- and mass-spectrometry-based analysis have revealed that IgAN patients showed elevated serum level of undergalactosylated IgA1 in O-linked glycans of its hinge region, called galactose-deficient IgA1 (Gd-IgA1). Thereafter, numerous studies have confirmed that the glomerular IgA from IgAN patients are enriched with Gd-IgA1; thus, the first hit of the current pathogenesis of IgAN has been considered to increase circulating levels of Gd-IgA1. Recent studies, however, demonstrated that this aberrant glycosylation alone is not sufficient to disease onset and progression, suggesting that several additional factors are required for the selective deposition of IgA in the mesangial region and induce nephritis. Herein, we discuss the current understanding of the characteristics of pathogenic IgA and its mechanism of inducing inflammation in IgAN.

## Introduction

Immunoglobulin A (IgA) is the most abundant isotype of antibodies (Abs); approximately 66 mg/kg IgA Abs is generated daily from antibody-secreting cells (ASCs) that reside mainly in the mucosal lumen (1, 2). There are two types of subclasses in human, namely, IgA1 and IgA2; the most significant structural difference between IgA1 and IgA2 is that IgA1 has a longer hinge region, while IgA2 lacks 13 amino acids compared to IgA1 (3). While approximately 90% of serum IgA is IgA1 subclass in its monomeric form, secretary IgA (sIgA), which are produced from plasma cells as dimeric or further polymeric forms, can be found predominantly in the mucosal lumen. The proportion of subclass of IgA is different depending on which segment of the mucosal lumen (4). The shorter hinge region of IgA2 than IgA1 makes it less susceptible to degradation by bacterial proteases, which would explain the higher prevalence of IgA2 in the lower gastrointestinal tract (GI). Monomeric IgA has an anti-inflammatory effector function, and sIgA (polymeric IgA) acts as neutralizing Abs against pathogenic virus or bacteria at mucosal surfaces (1, 5, 6). Therefore, IgA is generally considered as non-inflammatory Abs. However, IgA is sometimes involved in IgA-mediated diseases, including IgA nephropathy (IgAN) (7) and IgA vasculitis (8).

IgAN is the most common type of primary glomerulonephritis worldwide, with a global prevalence of 2.5 cases per 100,000 adults per year, and one of the first causes of end-stage renal disease (ESRD) (9). IgAN is characterized by the deposition of IgA and complement C3 in the glomerular mesangial region, often with co-deposition of IgG and/or IgM (10). Histologically, mesangial cell proliferation and expansion of extracellular matrix are observed (10). Although over half a century has passed since the first report of patients with IgAN by Berger et al. (11), no specific and causal treatment strategies have been developed, leading to ESRD in 30%–40% of cases within 10–20 years after disease onset (12). This is largely due to the lack of understanding of the pathogenesis of IgAN, particularly the characteristics of nephritogenic IgA. Herein, we summarize and discuss the current understanding of the characteristics of pathogenic IgA and its mechanism of inducing inflammation in IgAN.

## Characteristics of nephritogenic IgA in IgAN

In the 1980s, characteristics of IgA Abs deposited in glomeruli in IgAN began to be investigated. Monteiro et al. examined the mesangial IgA eluted from glomeruli of percutaneous renal biopsies of 20 patients with IgAN and directly demonstrated that mesangial IgA are predominantly polymeric and anionic (13). In early 2000s, the lectin- and mass-spectrometry-based analysis have revealed that IgAN patients showed elevated serum level of aberrantly glycosylated, specifically galactose-deficient, IgA1 in *O*-linked glycans of its hinge region (galactose-deficient IgA1: Gd-IgA1) (14, 15); more than 70% of patients with IgAN showed increased serum Gd-IgA1 levels above the 90th percentile of that in healthy controls (16). The amino acid sequence of the human IgA1 hinge region is proline-rich and consists of nine serine/threonines. *O*-Glycosylation usually occurs in three to six of these sites (17). B cells from IgAN patients have been reported to show decreased levels of core 1 β1,3-galactosyltransferase, the enzyme that attaches galactose to *N*-acetylgalactosamine (GalNAc) and molecular chaperon (Cosmc), which is necessary for galactosyltransferase stabilization (18). As a result, serum IgA1 with decreased galactose in *O*-linked glycans is increased in patients with IgAN. Thereafter, numerous studies have demonstrated that the glomerular IgA from IgAN patients are enriched with these galactose-deficient IgA1 (Gd-IgA1). This was further confirmed by the results of immunofluorescent (IF) staining of the kidney using KM55, an anti-Gd-IgA1 monoclonal Abs that we recently established (19, 20). Thus, Gd-IgA1 have been thought to play a central role in the pathogenesis of IgAN, although other glycosylation patterns of IgA1 have been reported, including the reduced sialylation of *O*-glycans and modified *N*-glycosylation of IgA1 (21).

This aberrant glycosylation in IgA alone, however, cannot be sufficient for the selective deposition of IgA in the mesangial region. In this regard, Berthelot et al. have reported that transferrin receptor 1 (TfR1) and transglutaminase 2 (TG2) expressed on mesangial cells are important for the IgA binding to the mesangial region (22). Meanwhile, Zeng et al. have reported the possibility that J chain expressed in mesangial cells might be involved in the selective deposition of IgA in the mesangial region (23). They performed a single-cell RNA sequencing using cells from the renal biopsies of 13 IgAN patients and normal tissues from six patients who had undergone nephrectomy and found significant upregulation of *JCHAIN* in mesangial cells. However, they have not directly demonstrated that J chain is involved in selective IgA deposition in the mesangial region, and it is unclear whether J chain expression precedes IgA deposition. Although the region-specific Ab deposition implies auto-Ab recognition of self-antigen(s), IgA-type auto-Abs against self-antigen in mesangial cells have not yet been identified. Therefore, the detailed mechanism by which IgA is specifically deposited in the mesangial region in patients with IgAN remains unclear.

## Origin of Gd-IgA1-involvement of mucosal immunity

In the pathogenesis of IgAN, the involvement of Gd-IgA1 production and mucosal immunity has been discussed (24, 25). This is because some of patients with IgAN show gross hematuria and exacerbation of nephritis after upper respiratory tract infection or colitis (10), and polymeric IgA containing J chains, generally found in the mucosa lumen, were detected in glomeruli from patients with IgAN (26). Moreover, recent genome-wide association studies provided the evidence for an association between IgAN and genes related to mucosal IgA production (*LIF*, *OSM, TNFSF13*, and *DEFA*) (27), deducing the connection between Gd-IgA1 production and mucosal immunity. However, it is still inconclusive where Gd-IgA1-producing cells are generated. Plasma cells in nasal-associated lymphoid tissue (NALT) predominantly produce IgA1 subclass (IgA1:IgA2 = 9:1), whereas those derived from gut-associated lymphoid tissue (GALT) generate a high amount of IgA2, although the IgA1/IgA2 ratio differs depending on which segment of the GI tract. Given that the subclass of IgA deposited in the kidney in IgAN is IgA1, it is speculated that Gd-IgA1 is derived from NALT. This theory is supported mainly in Japan, where tonsillectomy has been used to treat IgAN and showed clinical efficacy (28). However, the report from a subanalysis of the VALIGA study showed no efficacy of tonsillectomy in European patients with IgAN (29). Thus, tonsils are currently considered to be involved in the pathogenesis of IgAN only in a limited group of patients, such as Japanese subjects. On the other hand, in IgAN in Caucasian, the involvement of GALT in the production of Gd-IgA1 is strongly supported (24, 30). In Europe, there are relatively many reports showing the association of IgAN and celiac disease (31, 32) or inflammatory bowel disease (IBD) (33). The recent NEFIGAN Trial using intestinal selective steroids (Nefecon) in patients with IgAN, which was reported to reduce proteinuria in the treatment group (34), also supports the involvement of GALT in the pathogenesis of IgAN in Caucasian. Thus, the responsible mucosa that produces Gd-IgA1 may differ in different ethnicities. Details on the association of Gd-IgA1 production and mucosal immunity are described elsewhere (24, 25).

## Involvement of TLR in production of Gd-IgA1

Since mucosal IgA are generated by commensal bacteria, it is conceivable that certain bacteria are involved in Gd-IgA1 production. Although several intestinal or oral bacteria have been reported to affect clinical parameters in patients with IgAN (35, 36), specific bacteria directly involved in Gd-IgA1 production have not been identified. Instead, several types of toll-like receptor (TLR), a family of pattern recognition receptors that recognize pathogen-associated molecular patterns and play a central role in sensing microbial infection, have been reported to be involved in the pathogenesis of IgAN (24). Coppo’s group reported that TLR4 expression in circulating mononuclear cells from patients with IgAN was correlated with proteinuria (37), whereas Yu’s group recently showed that TLR7 expression was significantly increased in peripheral blood B cells from patients with IgAN (38). We have reported that TLR9, which recognizes unmethylated CpG motifs abundant in bacterial DNA, is involved in the production of abnormal glycosylated IgA. This is evident from our studies using IgAN model mice. We have shown that the ddY mouse strain, spontaneous IgAN model mice, could be classified into three groups based on the timing of disease onset: early-onset, late-onset, and quiescent groups. We performed a genome-wide scan of the early- and late-onset ddY mice and found that *MyD88*, an adaptor protein required for signaling of some TLRs, was a candidate gene for progression of renal injury. Analysis of mRNA expression of TLRs associated with MyD88 in splenocytes from ddY mice revealed a significant increase in TLR9 gene (39). *In vivo*, intranasal and systemic administration of CpG DNA into early-onset ddY mice aggravated nephritis (39, 40), suggesting that activation of TLR9 is involved at least in the pathogenesis of mouse IgAN. In human IgAN, we reported that polymorphisms of the TLR9 gene are associated with histological severity of patients with IgAN (39), indicating the involvement of TLR9 in the pathogenesis of human IgAN. Which types of TLR are involved in Gg-IgA1 production needs to be further investigated.

## Mechanism of glomerular inflammation

**Figure 1:**
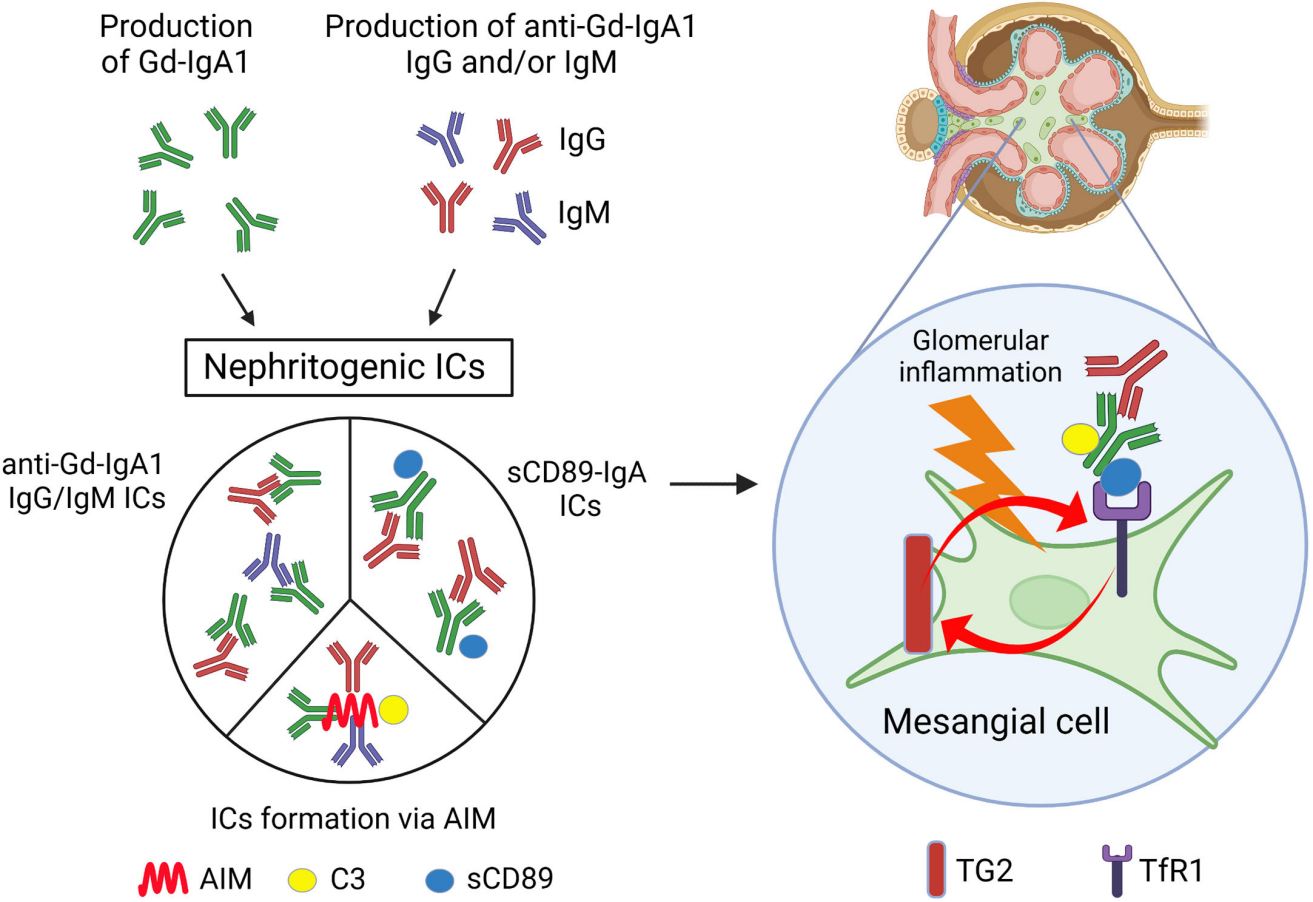
Scheme of the possible mechanism for glomerular inflammation in IgAN. Patients with IgAN show increased levels of serum Gd-IgA1 and anti-Gd-IgA1 IgG and/or IgM Abs. Nephritogenic ICs including these Abs, generated by various mechanisms as depicted, deposit mesangial region in glomeruli and induce mesangial inflammation. Gd-IgA1, galactose-deficient IgA1; ICs, immune complexes; AIM, apoptosis inhibitor of macrophages; sCD89, soluble fragment of CD89; C3, complement C3; TG2, transglutaminase 2; TfR1, transferrin receptor 1. Created with BioRender.com.

The IgA deposition in mesangial region is a hallmark of IgAN; however, the deposition of IgA alone is not sufficient for the subsequent glomerular inflammation. It was reported that renal transplant donors and necropsy individuals who died from traumatic injuries with glomerular IgA deposition were asymptomatic (41–43). Although these reports do not confirm that the glomerular IgA in these individuals were Gd-IgA1, it is likely that at least IgA deposition alone does not lead to induce nephritis. Hereafter, we will focus on the possible mechanism how IgA—generally considered as neutralizing Abs—induce glomerular inflammation in IgAN (**Figure 1**).

### Significance of IgA-containing nephritogenic immune complexes

In IgAN, immune complex (IC) formation is thought to be important in initiating glomerular inflammation. This has been experimentally demonstrated; both *in vitro* and *in vivo* experiments have shown that activation of human mesangial cells requires ICs containing IgG, not IgA alone (44, 45). The glomerular ICs in IgAN contain Gd-IgA1 and complement C3, IgG, and/or IgM. Although C3 is often co-deposited, IgG and IgM are not always detectable in routine IF microscopy. Recently, however, Rizk et al. have shown that IgG could be detectable even in the IgAN kidney-biopsy specimens without IgG by routine IF microscopy by extracting deposited Abs in glomeruli from remnant IgAN kidney-biopsy specimens (46), suggesting that IF microscopy using routine Abs is only under a sensitivity of detection, but in fact most patients have co-deposition of IgG. In the current widely accepted pathogenesis of IgAN, these IgG are thought to be auto-Abs that recognize Gd-IgA1 as a self-antigen. Tomana et al. reported that the reactivity of serum IgG extracted from IgAN patients to IgA1 was severely reduced when the *O*-linked glycans of the IgA1 hinge region was enzymatically removed, suggesting that sera from patients with IgAN contain IgG Abs against the *O*-linked glycans in the hinge region of IgA1 (47). In addition, we have reported that the affinity of patients’ IgG to IgA1 was enhanced when GalNAc was exposed by specifically removing sialic acid and galactose from the *O*-linked glycans of the IgA1 hinge region. Sequence analysis of the variable region of the heavy and light chains of these IgG revealed that the amino acid in the third position in the complementarity-determining region (CDR) 3 was serine in patients with IgAN rather than alanine in the healthy individuals (48). Further experiments revealed that this serine residue in CDR3 is required for effective binding to Gd-IgA1. These results suggest that endogenous anti-Gd-IgA1 IgG Abs recognize GalNAc as their antigen. It remains to be determined whether these IgG Abs are produced in an antigen (Gd-IgA1)-dependent manner or whether they are part of the existing natural Abs. Regarding IgM and IgG, Matsumoto et al. have recently demonstrated that anti-Gd-IgA1 IgM are present in circulating ICs from patients with IgAN, and these ICs contain high levels of complement C3 (49). These data may have identified one possible reason for the glomerular IgM co-deposition with IgA in IgAN. On the other hand, we demonstrated that ICs can be formed by mechanisms other than those described above, using gddY mice, an isolated group of ddY (grouped ddY: gddY) mice with a 100% incidence by selective mating of an early-onset group of ddY mice (50, 51). We have shown that the molecule apoptosis inhibitor of macrophages (AIM) is required to form ICs (52). We found that AIM accumulation in glomeruli colocalized with IgA in gddY mice and patients with IgAN. AIM-deficient (AIM^−/−^) gddY mice showed glomerular IgA deposition but no IgG, IgM, or C3 co-deposition and no proteinuria. Administration of recombinant AIM to AIM^−/−^ gddY resulted in IgG, IgM, and C3 co-deposition and subsequent proteinuria. These results suggest that ICs can be formed *via* AIM; AIM is responsible for binding glomerular IgA to other immunoglobulins and complement C3, although the mechanism is still unknown. In any case, through these mechanisms, Gd-IgA1 forms high-molecular nephritogenic ICs with IgG, IgM, and C3, thereby escaping clearance by the liver, the normal catabolic pathway for circulatory IgA1, and deposit in the glomeruli, causing subsequent inflammation in glomeruli.

### Soluble CD89 and transferrin receptor 1

IgA can interact with several receptors, including polymeric immunoglobulin receptor, TfR1 and FcαRI (CD89) (6). TfR was identified as IgA1 receptor expressed on human mesangial cells (53). CD89 is a member of the Fc receptor immunoglobulin superfamily and expressed on various blood myeloid cells including monocytes/macrophages, dendritic cells, neutrophils, and eosinophils (54). While CD89 binds monomeric IgA with a low affinity and generally exhibits anti-inflammatory functions following binding of serum monomeric IgA, CD89 binds polymeric IgA and IgA-antigen complexes with a higher avidity (55). Their binding induces phagocytosis (56), antibody-dependent cellular cytotoxicity (57), or release of inflammatory mediators (58). In IgAN, Monteiro et al. have reported the role of CD89 in the pathogenesis of IgAN. They found that circulating soluble fragment of CD89 (sCD89)-IgA complexes are present in adult patients with IgAN and further demonstrated that these complexes are involved in the pathogenesis of IgAN using the transgenic mice expressing human CD89 on macrophages and monocytes. In this mouse model, sCD89-IgA complexes were deposited in the mesangial region and induced glomerular inflammation (59). Moreover, recently, they reported that the levels of circulating sCD89-IgA complexes and free sCD89 were increased in childhood IgAN, and they were associated with proteinuria and MEST pathological score. In this report, they found that not only sCD89-IgA complexes but also sCD89 itself could directly induce mesangial cell proliferation both *in vitro* and *in vivo* (60). IgA-sCD89 ICs were deposited to glomeruli by sCD89 binding to TfR1 on mesangial cells (22). Gd-IgA1-sCD89 complexes may be easily deposited on mesangial cells, since IgA1 ICs and hypo-galactosylated IgA1 show higher affinity to TfR1 (61, 62). sCD89-TfR1 interaction has been shown to be impaired in the absence of TG2 (22), of which expression on glomeruli was related to the clinical and histological severity in IgAN patients (63). SCD89-IgA complexes–TfR1 interaction can be one of the explanations for the binding of IgA1 in the mesangial region in IgAN (**Figure 1**).

### Activation of complemental pathway

Complement activation has a pivotal role in triggering glomerular inflammation in IgAN (64). It occurs via three different pathways: the classical, the alternative, and the lectin pathways. In patients with IgAN, C3 and C4d mesangial depositions were observed in >90% and 18.9%–56%, respectively (64, 65), while C1q deposition is rarely observed (64). Glomerular C3 and C4d deposition were reported to be predictors of renal outcome in patients with IgAN (66, 67). Thus, complement activation in IgAN is considered to take place through the alternative and the lectin pathway. Although it was reported that IgA can activate both the alternative and the lectin pathway *in vitro* (68, 69), it is still unknown where Gd-IgA1 containing ICs activate the complement pathway, in circulation and/or after deposition in the mesangial region. In any case, the alternative and the lectin pathways lead to the formation of membrane attack complex (C5b-9) and initiate glomerular injury. In this process, anaphylatoxins C3a and C5a, which are produced by C3 activation, was also reported to be involved in glomerular inflammation (70, 71). Prolonged local inflammation resulting from these complement activations leads to chronic glomerular injury culminating in glomerulosclerosis.

## Conclusions and perspectives

Although IgA is generally considered as non-inflammatory Abs, however, IgA can induce IgA-mediated disease. Among them, IgAN is the most common. Although the key feature of nephritogenic IgA in IgAN is that they are under-galactosylated, the deposition of these IgA1 on glomeruli alone does not induce inflammation. Gd-IgA1 is likely to be generated via TLR (and/or other molecules) signaling and forms ICs with IgG, IgM, and complement C3 to induce nephritis; however, the detailed mechanism of these ICs formation remains unclear. Mesangial IgA deposition cannot solely be explained by the formation of these ICs containing Gd-IgA1, suggesting that other reasons for the selective deposition of IgA are present. Identification of the characteristics of such IgA might provide the key insights in the pathogenesis of IgAN.

## Author contributions

HS and YS were responsible for the conception and design of the review. YN and HS drafted and revised the manuscript. All authors contributed to the article and approved the submitted version.
